# A retrospective analysis of the protective efficacy of tafenoquine and mefloquine as prophylactic anti-malarials in non-immune individuals during deployment to a malaria-endemic area

**DOI:** 10.1186/1475-2875-13-49

**Published:** 2014-02-06

**Authors:** Geoffrey S Dow, William F McCarthy, Mark Reid, Bryan Smith, Douglas Tang, G Dennis Shanks

**Affiliations:** 1 United States Army Medical Materiel Development Activity, 1430 Veterans Drive, Fort Detrick, Maryland 21702, USA; 2 Clinical Network Services, 4/88 Jephson Street, Toowong, Queensland 4066, Australia; 3 Australian Army Malaria Institute, Weary Dunlop Drive, Gallipoli Barracks, Enoggera, Queensland 4051, Australia

## Abstract

**Background:**

In 2000/2001, the Australian Defense Forces (ADF), in collaboration with SmithKline Beecham and the United States Army, conducted a field trial to evaluate the safety, tolerability and efficacy of tafenoquine and mefloquine/primaquine for the prophylaxis of malaria amongst non-immune Australian soldiers deployed to East Timor (now called Timor Leste) for peacekeeping operations. The lack of a concurrent placebo control arm prevented an internal estimate of the malaria attack rate and so the protective efficacy of the study regimens was not determined at the time.

**Methods:**

In a retrospective analysis of the trial results, the all species malaria attack rate was estimated for the prophylactic phase of the study which was defined as the period between administration of the first prophylactic dose and the first dose of post-deployment medication. First, the *Plasmodium vivax* attack rate was estimated during the prophylactic phase of the deployment by adjusting the observed *P. vivax* relapse rate during post-deployment to account for the known anti-relapse efficacies (or effectiveness) of the study medications (determined from prior studies). The all species malaria attack rate (*P. vivax* and *Plasmodium falciparum*) was then determined by adjusting the *P*. vivax attack rate based on the ratio of *P. falciparum* to *P. vivax* observed during prior ADF deployments to Timor Leste. This estimated all species malaria attack rate was then used as the ‘constant estimated attack rate’ in the calculation of the protective efficacy of tafenoquine and mefloquine during the prophylactic phase of the deployment.

**Results:**

The estimated attack rate during the prophylactic phase of the study was determined to be 7.88%. The protective efficacies of tafenoquine and mefloquine, with corresponding 95% confidence intervals (95% CI), were determined to be 100% (93%-100%) and 100% (79%-100%) respectively.

**Conclusions:**

The protective efficacy of tafenoquine (200 mg per day for three days, followed by weekly 200 mg maintenance doses) is similar to that of the weekly standard of care (mefloquine, 250 mg).

## Background

Tafenoquine is a long elimination half-life [1] primaquine analog that has the potential to replace mefloquine for weekly malaria chemoprophylaxis. Tafenoquine was evaluated in a series of placebo-controlled studies in mixed or semi-immune residents of Africa and Southeast Asia in the late 1990s [2-4]. The weekly standard of care, mefloquine, was also evaluated in some of the same studies. The protective efficacies (95% CI) of tafenoquine at the intended dose (200 mg per day for three days followed by weekly 200 mg maintenance doses) and mefloquine in semi-immune residents of Ghana were 86% (76-92%) and 86% (72-93%) respectively [2]. In a second study in semi-immune residents of Kenya, the protective efficacy of tafenoquine was reported to be 86% (73-93%) [3]. For a discussion of the use of placebo control arms in malaria chemoprophylaxis studies in which semi-immune individuals are enrolled, see [5].

In 2000/2001, the safety, tolerability and efficacy of weekly tafenoquine and mefloquine were evaluated in malaria non-immune Australian soldiers from the 1^st^ Battalion, Royal Australian Regiment (1RAR) deployed on peacekeeping operations to Timor Leste and who volunteered to participate in a Phase 3 study [6]. In that study, which is referred to as Study 033 from here on, the protective efficacy of tafenoquine was not calculated because the study lacked a concurrent placebo control arm. A placebo control arm was not employed in Study 033 since the study volunteers were simultaneously participating in an ongoing peacekeeping operation and a placebo control arm was not considered appropriate in this context because malaria disrupts operational effectiveness. However, the study team concluded that both tafenoquine and mefloquine were effective since no malaria cases were observed during the prophylactic phase of the study, and the observation of *Plasmodium vivax* relapses during follow-up, together with epidemiology data from military and civilian sources suggested substantial exposure to malaria [7-10].

The United States Food and Drug Administration (FDA) [11] has stated: “The deployment of military personnel or civilian cohorts to malaria-endemic regions provides an opportunity to study anti-malarial prophylaxis in malaria-naive subjects. Since such deployments may last for many months, it is possible to standardize duration of malaria exposure. When placebo-controlled studies cannot be performed, well-characterized epidemiological attack rates can be used to calculate protective efficacy”. Furthermore, the FDA [11] also recommends that the calculation of protective efficacy in historical-controlled studies should employ epidemiological attack rates in the study area from at least the past two malaria seasons. In addition, these epidemiological attack rates should closely reflect anticipated attack rates in the study population and should be derived from the same geographical area, during the same seasonal period, with similar rainfall and similar subject exposure.

Malaria epidemiological data were collected from ADF personnel deployed to Timor Leste in the year prior to Study 033 [12]. That study reported an attack rate of ~4.8% across all deployed military personnel. These data do not completely meet the requirements of the FDA guidance [11] for two reasons. First, the data were collected only for a single year. Second, the operational environment changed between 1999/2000 and 2000/2001, meaning that we do not know whether exposure of ADF personnel to malaria remained constant. Malaria prevalence was measured amongst civilians who were not taking prophylaxis in villages adjacent to garrisoned Australian soldiers during Study 033 [7]. Similar data are not available for the prior year, so cannot be used to directly link exposure in Australian soldiers in the two deployments.

Immunologic markers of malaria exposure continue to be explored as a possible surrogate for observed malaria cases in malaria prophylaxis studies in which a placebo control arm was not used. Antibodies to circumsporozoite protein were measured as a secondary endpoint in the atovaquone-proguanil clinical development program [13]. However, in that study, seroconversion was only 1.1%, and since there were no confirmed symptomatic cases, it is unclear to what extent this reflects true exposure. The US Army recently investigated the possibility that antibodies to merozoite surface protein 1 (MSP1) might serve as a surrogate marker of malaria exposure in non-immune individuals given mefloquine for prophylaxis [14]. Regulatory agencies have not yet embraced the use of such surrogates [11], although it is possible they might if more compelling evidence of their utility became available. In any case, since they are not currently available, a retrospective analysis of stored blood samples, even if feasible, would likely not reveal useful information regarding malaria exposure during Study 033.

Because of the limitations stated above, an alternative approach was needed to satisfy the FDA’s requirement that a well-characterized epidemiological attack rate should be used to calculate protective efficacy when a placebo control arm is not appropriate. To this end, the *P. vivax* attack rate during the prophylactic phase of Study 033 in Timor Leste was estimated through adjustment of the observed post-deployment *P. vivax* relapse rate during the follow-up period of the study to account for the known anti-relapse efficacies (or effectiveness) of the study medications. This methodological separation between what would have been primary *P. vivax* cases during deployment and post-deployment relapses is reasonable, because, while both events are triggered by the same initial exposure, they are independent clinical episodes prevented through independent pharmacologic modalities that pose different risks to the individual and deployed force. An all malaria attack rate was estimated by adjusting the *P. vivax* attack based on the ratio *Plasmodium falciparum* to *P. vivax* cases observed amongst Australian soldiers in Timor Leste a year prior to Study 033. The estimated all malaria attack rate was then utilized to determine, retrospectively, the protective efficacy of both tafenoquine and mefloquine in Study 033 so a comparison could be made to the placebo controlled studies in semi and mixed immune populations.

## Methods

### Definition of prophylactic efficacy

For the purposes of this study the definition of protective efficacy is the same as was used in the original Study 033 protocol: Ability of study medications with recorded administration to prevent clinical malaria (single positive smear of any species with concurrent signs and symptoms consistent with malaria infection) during prophylactic study drug administration up to and including the first dose of post-deployment medication.

### Definition of anti-relapse efficacy/effectiveness

For the purposes of this study, anti-relapse efficacy/effectiveness is the degree to which observed (or unsupervised) tafenoquine and primaquine administration can prevent *P. vivax* relapses. There was no distinction made here between the intent or possible timing of 8-aminoquinoline administration, whether it be following a course of suppressive prophylaxis, an additional property of an anti-malarial drug also being used in a prophylactic mode, or in sequential combination with a blood schizonticidal drug to treat symptomatic *P. vivax*. In the case of historical estimates of the utility of primaquine, we have used a meta-analysis of effectiveness data [15], since many of the input studies utilized unsupervised primaquine regimens. The corresponding tafenoquine data represent the efficacy of the drug since the studies from which they were derived involved observed use of the drug.

### Primary *P. vivax* attacks during deployment and post-deployment relapses are not the same

Post-deployment *P. vivax* relapses are not the same as primary *P. vivax* attacks occurring during deployment because while triggered by the same initial exposure, are independent clinical events prevented through independent pharmacologic modalities and which pose different risks to the individual and deployed forces.

During a visit to a malarious area, non-immune individuals exposed to *P. vivax* will experience a primary symptomatic attack and if treated with anti-malarial drugs to kill asexual blood stages may still go on to experience one or more hypnozoite-intiated relapses. To prevent such primary attacks during travel or deployment it is conventional to administer a prophylactic that inhibits the development of asexual blood stages or exoerythrocytic stages (or both) in the liver. Since these properties of a prophylactic drug do not prevent the establishment of the *P. vivax* hypnozoite, many individuals who receive such a drug will experience one or more post-deployment relapses rather than a primary attack.

Independent post-deployment administration of primaquine substantially reduces, but not to zero, the number of *P. vivax* relapses due to the effect of the drug on hypnozoites [15,16]. This anti-hypnozoite activity is mediated independently of the other effects of primaquine on asexual blood and developing exoerythrocytic stages. The same logic holds for tafenoquine, since the drug exhibits a measurable inhibitory effect on *P. vivax* hypnozoites independent of its other actions on asexual blood stages and developing exoerythrocytic parasites in the liver [4,17,18].

Based on the World War II experience with malaria in the Pacific theatre [19], it seems intuitive that the management of a small number of individuals with *P. vivax* hypnozoites refractory to primaquine (or tafenoquine) is more straight-forward and has less operational impact than a larger number of primary attacks in theatre (in the absence of effective prophylaxis). In contrast, an individual who takes effective prophylaxis without an 8-amino-quinoline is at greater risk of repeated post-deployment *P. vivax* relapses despite having being exposed only once and never experiencing a primary attack. Furthermore, the clinical impact of a primary attack on a traveller may be greater than a relapse in their own country if they do not have access to good medical care while travelling.

Therefore, the deployment prophylactic and anti-relapse efficacies of proposed interventions can and should be measured independently. As a first step towards calculation of the former, an estimate of the attack rate is required. In this study, since the post-deployment relapse rate is known, and the anti-relapse efficacy of the combined study regimens can be estimated, it is possible to estimate the number of individuals exposed to *P. vivax* during a deployment.

### Summary of study 033

From October 2000 through April 2001, the 1RAR was deployed to Timor Leste from Australia for peacekeeping operations [6]. Six hundred and fifty four of these soldiers volunteered to participate in Study 033, and were randomized to receive either tafenoquine (n = 492) or mefloquine (n = 162) for malaria prophylaxis. Tafenoquine was administered as a loading dose of 200 mg once per day for three days, followed by weekly 200 mg maintenance doses. Mefloquine was administered as a 250 mg loading dose once per day for three days followed by weekly 250 maintenance doses. During this prophylactic phase of the study, the primary efficacy parameter was the occurrence of microscopically confirmed symptomatic malaria of all species.

Upon return to Australia, subjects randomized to mefloquine and tafenoquine received either 15 mg primaquine twice per day for 14 days (total dose of 420 mg or 5.2 mg/kg) or placebo. There was a six month period of follow up. The intent during this phase was to monitor *P. vivax* relapses. Primaquine was given in the mefloquine arm because it is an effective anti-relapse agent and mefloquine is not. A placebo was given in the tafenoquine arm because prior Phase II studies suggested it was also an effective anti-relapse agent.

During the prophylactic phase of the study, there were no symptomatic cases of malaria in either study arm. During the six-month follow-up phase there were four *P. vivax* relapses in the tafenoquine arm and one *P. vivax* relapse in the mefloquine arm.

In the final clinical study report (FCSR) subsequently submitted to the FDA by the Sponsor, the intent to treat (ITT) populations were n = 490 for tafenoquine, and n = 161 for mefloquine. The ITT denominators were used for the attack rate and protective efficacy calculations that follow. The reader should also note that in the FCSR submitted to the FDA by the Sponsor, 21 subjects were reported as being withdrawn from the study. The Sponsor conducted a "worst case analysis" for the intention to treat (ITT) population. Any subject that withdrew during the prophylactic phase was considered a failure (assumed to have had malaria). This "worst case analysis" for the ITT population yielded a protective efficacy, assuming a 100% attack rate, of 96.5% (473/490) and 97.5% (157/161) for tafenoquine and mefloquine, respectively. The assumption that withdrawn subjects were prophylactic failures is the most conservative approach for such an ITT efficacy analysis. Therefore, an important component of the present study was to confirm that the individuals who withdrew from the study should not be counted as prophylactic failures due to loss to follow-up. This issue was not explicitly addressed in the original public reporting of the study results [6].

### ADF deployments to Timor Leste

Australian Defense Force infantry battalions commenced peacekeeping operations in Timor Leste in September 1999 as part of International Force East Timor (InterFET) under United Nations Security Resolution 1264 and with Indonesian government agreement. Table 1 summarizes the time period ADF infantry battalions served in Timor Leste up to 30 April 2003. Later battalions starting with the 5^th^/7^th^ Battalion, Royal Australian Regiment (5/7RAR) in October 1999 migrated to operations under the United Nation’s Transitional Administration East Timor (UNTAET, Security Resolution 1272 in 1999). In contrast to ADF soldiers under InterFET, those deployed under UNTAET experienced less exposure to night jungle patrols compared to previous operations in multiple regions of Timor Leste; this may have contributed to reduced vector exposure. Vector control operations also improved as lines of communication between Australia and Timor Leste improved; soldiers gradually re-learned basic malaria prevention procedures in infantry units over time.

**Table 1:** Details of Australian infantry deployments to Timor Leste*

**Battalion**	**Dates**	**Regions of Timor Leste**	**Battalion strength**	**Months deployed**	Pf attack rate (%) Monthly/Cumul ***	**Pv attack rate (%) Monthly/Cumul**	**All malaria attack rate (%) Monthly/Cumul**	**Prophylaxis**
2 RAR**	Sep 99 through Jan 00	Dili, Bobanaro district	681	4.33	0.31/1.32	2.04/8.81	2.34/10.13	Doxycycline 100 mg q.d
3RARⱡ	Sep 99 through Jan 00	Dili, Bobanaro district and Oecussi province	634	4.33	0.29/1.26	2.11/9.15	2.59/11.2	Doxycycline 100 mg q.d
5/7RAR	Nov 99 through Apr 00	Dili, Bobanaro district	522	6.83	0.11/0.77	0.81/5.56	0.83/6.71	Doxcycline 100 mg q.d
6RAR	Apr 00 through Oct 00	Dili, Bobanaro district	619	6.16	0.03/0.16	0.24/1.45	0.26/1.62	Doxycycline 100 mg q.d
1RAR	Oct 00 through Apr 01	Dili, Bobanaro district	723	6.00	0.00/0.00	0.18/1.11	0.18/1.11	Tafenoquine 200 mg or Mefloquine 250 mg weekly
4RAR	Apr 01 through Oct 01	Dili, Bobanaro district	750	7.00	0.00/0.00	0.08/0.53	0.08/0.53	Mefloquine 250 mg weekly
2RAR	Oct 01 through May 02	Dili, Bobanaro district	681	6.83	0.00/0.00	0.11/0.73	0.11/0.73	Mefloquine 250 mg weekly
3RAR	Apr 02 through Oct 02	Dili, Bobanaro district	634	7.00	0.02/0.16	0.14/0.95	0.18/1.26	Doxycycline 100 mg q.d
5/7RAR	Oct 02 through Dec 02	Dili, Bobanaro district	536	2.57	0.00/0.00	0.28/0.71	0.28/0.71	Doxycycline 100 mg q.d

*Values for dates deployed, battalion strength and period deployed are approximate.

**RAR = Royal Australian Regiment.

ⱡBoth 3 RAR and 5/7 RAR soldiers participated in post-exposure prevention studies (Elmes, 2008).

***Monthly attack rate (%) = (Cases/total person-months) x 100; cumulative attack rate (%) = (cases/strength) x 100.

### Documentation of malaria exposure during ADF deployments to Timor Leste

Malaria is a notifiable disease in all states and territories of Australia under the National Notifiable Disease Surveillance System [20]. Australian Defence Force Health Policy Directive 215 requires notification of malaria infections in military personnel to the state or territory health authority. In addition, all confirmed or suspected cases of malaria in defense personnel or their dependents (whilst on posting to malarious areas) are reported to the Australian Army Malaria Institute (AMI) Central Malaria Registry (CMR). Whenever possible all malaria diagnosis is achieved with light microscopy of thick and thin blood smears. Diagnosis may also be performed using malaria rapid diagnostic tests (RDTs) when accurate diagnosis by microscopy is not available. The notification report to AMI by defence units is accompanied by thick and thin blood films as well as any RDT cards or other devices used to diagnose malaria to allow AMI personnel to verify the diagnosis and species before entry into the CMR.

The CMR was analysed over the period 1 April 1999 to 30 April 2003 for malaria cases in the infantry battalions described in Table 1. Malaria cases were excluded if the infantry soldier had a travel history to another malarious country after leaving Timor Leste and their first confirmed case of malaria. All *P. vivax* relapses were excluded from the analysis irrespective of country of origin. Duplicates entries (total of 17) in the CMR database were confirmed against the original notification and follow-up relapse reports (if applicable) and subsequently discounted. A limitation of the analysis is loss to follow-up of Defense members who leave the ADF and subsequently develop malaria after a military deployment. These cases would be captured by state/territory health authorities but not necessarily by the ADF unless a compensation action was commenced. The analysis was subjected to an independent quality control review. All work complied with the National Statement on Ethical Conduct in Human Research (as amended) guidance on the use of identifiable databanks [21].

### Determination of the malaria status of subjects who withdrew from Study 033

As mentioned earlier, the FCSR report submitted to the FDA by the Sponsor included a "worst case analysis" for the ITT population in which subjects who withdrew from the study were considered prophylactic failures. In the reanalysis of Study 033, there was an attempt to confirm that subjects who withdrew were not in fact prophylactic failures (i.e. did not get malaria) if they remained in the ADF for up to 12 months post their 1 RAR deployment and had no other travel history to malarious areas. Three investigators queried the CMR to determine the malaria status of each of these subjects. As mentioned above, a limitation of the CMR is that it captures malaria cases only for active ADF personnel. Additional ADF records were examined to determine whether any of the subjects withdrawn from the study left the ADF in the twelve months following the end of Study 033.

### Observed post-deployment *P. vivax* relapse rate during Study 033

The number of *P. vivax* cases from study subjects enrolled in Study 033 in the CMR was determined during a period of one year post-deployment. This observation period was longer than that in the Study 033 final clinical study report (6 months) because Chesson strain *P. vivax* has been recorded as presenting in the ADF for up to 505 days after the first day of deployment (median 83 days, mean 105 days). As a consequence more *P. vivax* cases were recorded than were reported previously (8 versus 5). The 12 month observed post-deployment *P. vivax* attack rate [AR_Pv_ (post-deployment)] was then calculated as follows (equation 1):

(1) $$\begin{array}{l} AR_{\mathrm{Pv}}\;\left(12\;\mathrm{month}\;\mathrm{post}‒\mathrm{deployment}\right)=\left(\frac{R_{\mathrm{Pv}}}{N}\right)100\%\\ \;\;\left[\left(\frac{8}{651}\right)100\%=1.23\%\right] \end{array}$$

Where R_Pv_ = total number of *P. vivax* relapses and N = total ITT sample size

### Calculation of the ratio of *P. falciparum* to *P. vivax* cases during the InterFET deployment (1999/2000)

The ratio of cases of *P. falciparum* to *P. vivax* was estimated as described below and summarized in Table 2 (A-D). The 1RAR soldiers involved in the 2000/01 ADF deployment and who were enrolled in Study 033 arrived in country on 25 October 2000 and returned to Australia on approximately 25 April 2001. This coincided with the wet season and the period of highest seasonal malaria prevalence in Timor Leste [9]. Three separate battalions of Australian soldiers were deployed at times that overlapped this period the year before; Second Battalion, Royal Australian Regimen (2RAR), Third Battalion, Royal Australian Regiment (3RAR) and the 5/7RAR. Their malaria exposure was assumed to be the most relevant because it also coincided with period of highest seasonal malaria prevalence. A fourth, the 6^th^ Battalion, Royal Australian Regiment (6RAR), was deployed in the six months prior to 1RAR in which conditions were drier, and in which the incidence of malaria is usually lower [9]. Together, the 2RAR, 3RAR, and 5/7 RAR were deployed for three periods of overlapping exposure which were relevant. Period 1 was October 25 to October 31 1999, and includes only 2RAR and 3RAR since 5/7 RAR was not yet deployed. Period 2 was from November 1 1999 to January 31 2000 and included 2RAR, 3RAR and 5/7RAR. Period 3 was from February 1 2000 to April 25 2000 and includes only 5/7 RAR since the other battalions had returned to Australia.

**Table 2:** **(A-D): Calculation of the 6 month cumulative attack rate ( *Pf*, *Pv*, all species) and *Pf to Pv *** **ratio during the 1999/00 ADF deployment**

				
A: Cumulative attack rates (%) by time period, species and battalion exposed during the 1999/2000 ADF deployment*				
Time period (months)/(Battalions deployed)	Species	2RAR (n = 681)	3RAR (n = 634)	5/7RAR (n = 522)
Period 1: Oct 25 99-Oct 31 00 (0.2)/(2RAR and 3RAR)	Pv/Pf/All species	0.41/0.06/0.47	0.44/0.07/0.52	NA/NA/NA
Period 2: Nov 1 99-Jan 31 00 (3.0)/(2RAR, 3RAR and 5/7 RAR)	Pv/Pf/All species	6.10/0.92/7.02	6.56/0.98/7.76	2.52/0.34/2.95
Period 3: Feb 1 00 – April 25 00 (2.8)/(5/7RAR)	Pv/Pf/All species	NA/NA/NA	NA/NA/NA	2.38/0.32/2.78
B: Estimate of overall cumulative attack rate (%) by period for ALL battalions exposed during the 1999/2000 ADF deployment*				
Time period (months)	Species		Cumulative attacks for all battalions	
Period 1: Oct 25 99-Oct 31 99 (0.2)	Pv/Pf/All species		0.42/0.06/0.49	
Period 2: Nov 1 99-Jan 31–00 (3.0)	Pv/Pf/All species		5.24/0.77/6.12	
Period 3: Feb 1 00 – April 25 00 (2.8)	Pv/Pf/All species		2.38/0.32/2.78	
C: Estimate of the 6 month cumulative attack rate for battalions exposed during the 1999/00 ADF deployment**				
Time period (months)	Species		Cumulative attack rates for all battalions	
Periods 1, 2 and 3:Oct 25 99-April 25 00 (6.0)	Pv/Pf/All species		7.89/1.15/9.18	
D: Estimate of Pf to Pv ratio for all battalions exposed during the 1999/00 ADF deployment***	0.146 (1.15/7.89)			

*Overall attack rates are the weighted averages of attack rates for battalions exposed during the period (weights = sample size).

**Cumulative attack rate (probability) = P1 + (1-P1)*P2 + (1-P1)(1-P2)*P3 where P1, P2 and P3 are the estimated overall attack rates for periods 1, 2 and 3 (from Table 2B).

***Cumulative Pf attack for all battalions (from Table 2C)/Cumulative Pv attack rate for all battalions (from Table 2C).

Table 2A summarizes for each period and regiment, the *P. falciparum*, *P. vivax*, and all species malaria attack rates (AR) calculated as follows (equation 2):

(2) $$\begin{array}{l} \mathrm{AR}{\left(\mathrm{in}\;\mathrm{period}\right)}_{\mathrm{Pv},\mathrm{Pf},\mathrm{all}}=\left[\frac{{\left(\mathrm{total}\;\mathrm{cases}\right)}_{\mathrm{Pv},\mathrm{Pf},\mathrm{all}}}{\left(\mathrm{person}‒\mathrm{months}\;\mathrm{deployment}\right)}\right]\\ \;\;\;\;\times \left(\mathrm{months}\;\mathrm{in}\;\mathrm{period}\right)100\% \end{array}$$

During each of these three time periods, an overall (weighted average) attack rate was calculated with weights based on the number of soldiers in each battalion (Table 2B). Using the overall attack rates (decimal) for each period as estimates of the malaria risk (probability), the estimate of the cumulative 6 month attack rate [((AR(6 month), Table 2C) for each species and all species (*P. vivax* and *P. falciparum*) were obtained as follows (equation 3):

(3) $$\begin{array}{l} \mathrm{AR}\;{\left(\mathrm{six}\;\mathrm{month}\right)}_{\mathrm{Pv},\mathrm{Pf},\mathrm{all}}=AR_{P1}+\left(1-AR_{P1}\right)AR_{P2}\\ \;\;\;\;+\left(1-AR_{P1}\right)+\left(1-AR_{P2}\right)AR_{P3} \end{array}$$

The *P. falciparum* to *P. vivax* ratio (*Pf:Pv*) was then determined (Table 2D) as follows (equation 4):

(4) $$\mathrm{Pf}:\mathrm{Pv}=\;\frac{\mathrm{AR}{\left(6\;\mathrm{month}\right)}_{\mathrm{Pf}}}{\mathrm{AR}{\left(6\;\mathrm{month}\right)}_{\mathrm{Pv}}}\;\left[\frac{1.15}{7.89}=0.146\right]$$

### Assumed anti-relapse efficacy/effectiveness of Study 033 regimens

For estimation of the *P. vivax* attack rate during the prophylactic phase, it was assumed that the anti-relapse efficacy/effectiveness of the combined (ARE_combined_) post-exposure prophylaxis regimens (tafenoquine or primaquine) was 82.1%. This was obtained as a weighted average of estimates of the anti-relapse efficacy/effectiveness of tafenoquine and primaquine and was calculated as follows (equation 5):

(5) $$\begin{array}{l} \mathrm{AR}E_{\mathrm{combined}}=\left(\frac{N_{\mathrm{Tq}}\left(\mathrm{AR}E_{\mathrm{Tq}}\right)+N_{\mathrm{Mef}}\left(\mathrm{AR}E_{\mathrm{Pq}}\right)}{\left(N_{\mathrm{Tq}}+N_{\mathrm{Mef}}\right)}\right)100\%\\ \;\;\;\left[\left(\frac{490\left(0.863\right)+161\left(0.695\right)}{\left(490+161\right)}\right)100\%=82.1\%\right] \end{array}$$

Where N_Tq_ and N_Mef_ are the ITT sample sizes in the post-exposure tafenoquine and mefloquine arms of Study 033 and ARE_Tq_ and ARE_Pq_ are the anti-relapse efficacies/effectiveness of tafenoquine and primaquine determined from published studies as outlined in the following paragraphs.

ARE_Pq_ was based on pooled data from a number of historical studies in which the relapse rate in a chloroquine only arm (26.5%; 420/1585) was compared with chloroquine + 2.5- < 5 mg/kg primaquine (8.08%; 140/1732 ) as reported by [15]. Based on these relapse rates, the assumed ARE_Pq_ is 69.5% ([(0.265-0.0808)/0.265]100%). Note that this represents the effectiveness of primaquine in this context because administration of the drug in many of the studies analysed by John et al. (2012) was not directly observed.

The assumed anti-relapse efficacy of tafenoquine (ARE_Tq_) was based on data reported by Walsh *et al.*[4]. Walsh *et al.* investigated the prophylactic efficacy of tafenoquine administered as a loading dose of 400 mg × 3, followed by monthly maintenance doses of 400 mg for five months versus placebo in deployed Thai soldiers. *P. vivax* relapses were observed in 3.1% (3/96) of subjects randomized to tafenoquine and completing the study, where as 22.8% (21/92) of subjects randomized to placebo who completed the study contracted *P. vivax* in the prophylactic phase [4]. In that study, subjects in the placebo control arm who contracted malaria were administered a curative regimen and recruited for a subsequent tafenoquine pharmacokinetic study, meaning that the *P. vivax* relapse rate in this arm is not known. It was assumed the *P. vivax* relapse rate in that study was 100%. Therefore, the assumed anti-relapse rate of tafenoquine was 86.3% ([(0.228 – 0.031)/0.228]100%).

### Estimated malaria attack rate during Study 033

The estimated all malaria attack rate during the six-month prophylactic phase of Study 033 was calculated as the sum of the estimated six month attack rates for *P. vivax* and *P. falciparum* as follows (equation 6).

(6) $$\begin{array}{l} \mathrm{AR}{\left(\mathrm{prophylaxis}\;\mathrm{phase}\right)}_{\mathrm{all}}\\ \;\;=AR_{\mathrm{Pv}}+AR_{\mathrm{Pf}}\;\left[6.88\%+1.00\%=7.88\%\right] \end{array}$$

The calculations are further summarized in Table 3. Note that species other than *P. vivax* and *P. falciparum* were excluded.

**Table 3:** **Estimation of malaria attack (*Pv*, *Pf*, all species) during the prophylactic phase (6 month) of Study 033**

Data*	Value**	**Source**
Post deployment *P. vivax* relapse rate (%) amongst Study 033 subjects	1.23	*Pv* relapses (8/651) from CMR not original Study 033 FCSR (Equation 1)
Anti-relapse effectiveness (%) of primaquine	69.5	Estimated from John et al. 2012
Anti-relapse efficacy (%) of tafenoquine	86.3	Estimated from Walsh et al. 2004b
Anti-relapse efficacy of combined Study 033 post-exposure prophylaxis regimens	82.1	Estimated (Equation 5)
*Pv* attack rate (%) during prophylactic phase of Study 033	6.88	Estimated (Equation 7)
Ratio of *Pf* cases to *Pv* cases in 1999/2000 ADF deployment (InterFET)	0.146	Observed from 1999/2000 ADF deployment (Equation 4)
*Pf* attack rate (%) during prophylactic phase of Study 033	1.00	Estimated (Equation 8)
All malaria attack rate (%) during prophylactic phase of Study 033	7.88	Estimated (Equation 6)

*Observed in Study 033, ADF deployment, assumed from literature or derived. **Rounded *after* calculation.

The *P. vivax* attack rate (AR_Pv_) during the prophylaxis phase was estimated by adjusting the total observed relapse rate of 1.229% (8/651) during the post-deployment period to take into account the combined anti-relapse efficacy of 82.1% in the tafenoquine and mefloquine/primaquine arms (Eqn 5; Table 3) as follows (equation 7)

(7) $$\begin{array}{l} AR_{\mathrm{Pv}}\;\left(\mathrm{prophylaxis}\;\mathrm{phase}\right)=\frac{AR_{\mathrm{Pv}}\left(12\;\mathrm{month}\;\mathrm{post}‒\mathrm{deployment}\right)}{1‒\mathrm{AR}E_{\mathrm{combined}}}\\ \;\;\;\;\;\left[\frac{1.229\%}{1‒0.8213}=6.88\%\right] \end{array}$$

It was assumed that all *P. vivax* cases that would have been observed in the prophylactic phase would also have relapsed in the absence of post-exposure prophylaxis. The observed *P. vivax* relapse rate during the post-deployment period of Study 033 (1.229%) was determined using data from the CMR as described earlier.

The estimate of the *P. falciparum* attack rate during the six-month prophylaxis was based on the estimate of the *P. vivax* attack rate during the prophylaxis phase (6.88%; from equation 7) and the ratio of *P. falciparum* relative to *P. vivax* (0.146, from equation 4) based on epidemiological data from the 1999/00 ADF deployments to Timor Leste (Table 2) and calculated as follows (equation 8).

(8) $$\begin{array}{l} AR_{\mathrm{Pf}}\;\left(\mathrm{prophylaxis}\;\mathrm{phase}\right)=AR_{\mathrm{Pv}}\left(\mathrm{Pf}:\mathrm{Pv}\;\mathrm{ratio}\right)\\ \;\;\;\;\left[6.88\%\left(0.146\right)=1.00\%\right] \end{array}$$

### Calculation of the protective efficacy and confidence intervals for tafenoquine and mefloquine

The usual definition of the protective (prophylactic) efficacy (PE) in a placebo-controlled trial was used to determine the PE for tafenoquine (PE_Tq_) during the prophylaxis phase as follows (equation 9):

(9) $$PE_{\mathrm{Tq}}=\left(\frac{AR_{\mathrm{Pl}}-AR_{\mathrm{Tq}}}{AR_{\mathrm{Pl}}}\right)100\%=\left(1‒\frac{AR_{\mathrm{Tq}}}{AR_{\mathrm{Pl}}}\right)100\%$$

Where AR_Tq_ and AR_Pl_ are the cumulative risk estimates (cumulative attack rates) for the period of prophylaxis in those receiving tafenoquine compared to those receiving a hypothetical placebo. A similar definition applies to the protective efficacy of mefloquine (PE_Mef_). Since Study 033 had no placebo control arm, conventional calculation of protective efficacy and corresponding confidence interval (CI) was not possible. Because in Study 033 there were no cases of malaria it should be noted that, for any estimated attack rate, the point estimate PE_Tq_ and PE_Mef_ would be 100%. However, standard methods for calculating a confidence interval (CI) for PE (Tq or Mef) depend on the placebo attack rate and sample size. To determine the PE and CI it was assumed that the attack rate (AR_Pl_) would have been the estimated all malaria attack rate of 7.88% calculated above (equation 6) during the prophylactic phase of Study 033. In addition a constant AR_Pl_ was (= 7.88%) used for calculating corresponding 95% CIs for PE. A sensitivity analysis of this assumption was carried out (Table 4, see below). In the remainder of this discussion the estimated all malaria attack rate (=7.88%) will be referred to as the “estimated AR” (AR_Pl_).

**Table 4:** Effect of using a constant baseline attack rate on the precision (confidence interval) of the estimated protective efficacy of tafenoquine compared to assuming an attack rate based on results from hypothetical placebo controlled trials with different sample sizes

**AR**_**Pl **_**(cases/n)**^**1**^	**AR**_**Tq **_**(cases/n)**^**2**^	**PE**_**Tq**_	**LL (PE**_**Tq**_**)**^**3**^
1% (1/100)	0 (0/490)	100%	45% (44.87)
1% (10/1000)	0 (0/490)	100%	45% (44.91)
1% (constant)^4^	0 (0/490)	100%	44% (44.10)
2% (1/50)	0 (0/490)	100%	72% (72.43)
2% (2/100)	0 (0/490)	100%	72% (72.46)
2% (constant)^4^	0 (0/490)	100%	73% (72.54)
3% (3/100)	0 (0/490)	100%	82% (81.65)
3% (30/1000)	0 (0/490)	100%	82% (81.69)
3% (constant)^4^	0 (0/490)	100%	82% (81.70)
5% (1/20)	0 (0/490)	100%	89% (88.97)
5% (2/40)	0 (0/490)	100%	89% (88.98)
5% (5/100)	0 (0/490)	100%	89% (88.99)
5% (50/1000)	0 ( 0/490)	100%	89% (89.02)
5% (constant)^4^	0 (0/490)	100%	89% (89.02)
10% (1/10)	0 (0/490)	100%	94% (94.49)
10% (5/50)	0 (0/490)	100%	95% (94.50)
10% (10/100)	0 (0/490)	100%	95% (94.50)
10% (100/1000)	0 (0/490)	100%	95% (94.51)
10% (constant)^4^	0 (0/490)	100%	95% (94.51)

^1^Placebo attack rate based on hypothetical trial results (cases/n) or assumed to be a known constant value (no variability).

^2^Observed attack rate for tafenoquine in Study 033.

^3^Lower limit of the one-side 95% CI for the protective efficacy of tafenoquine [LL(PETq)]. Koopman's (score test) method was used when the placebo attack rate (AR_Pl_) is based on hypothetical placebo results (cases/n). StatXact (vs.9.0) was used for calculations and the confidence level was set to 90% [for ARTq = 0 (0/n) this gives a lower limit of a one-sided 95% interval for PE_Tq_].

^4^When AR_Pl_ is assumed to be a known constant equation 11(bottom) was used to obtain LL(PE_Tq_).

The attack rate for tafenoquine (AR_**Tq**_) and mefloquine (AR_Mef_) during the prophylactic phase was determined based on the ITT results of study 033 [0 cases; sample size of 490 (Tq) and 161 (Mef)] and were computed as follows (equation 10):

(10) $$\begin{array}{l} AR_{\mathrm{Tq},\mathrm{Mef}}\left(\mathrm{prophylaxis}\;\mathrm{phase}\right)\;=\frac{{\left(\mathrm{All}\;\mathrm{malaria}\;\mathrm{cases}\right)}_{\mathrm{Tq},\mathrm{Mef}}}{N_{\mathrm{Tq},\mathrm{Mef}}}\\ \;\;\;\;\;\left[\frac{0}{490},\frac{0}{161}\right] \end{array}$$

The 95% CI for PE_Tq_ (PE_Mef_) was obtained by first computing the lower and upper limits (LL, UL) for a proportion (AR_Tq_ or AR_Mef_) using the Wilson (score) method, and then converting these limits into corresponding limits for PE (assuming AR_Pl_ = 7.88%). When there are 0 cases, AR = 0 (0/n) and the lower confidence limit for AR is always 0 and the corresponding upper limit for PE is 100%. Given lower and upper Wilson confidence limits for the attack rates (LL _AR_, UL_AR_) the corresponding limits for PE (Tq or Mef) are therefore given as follows (equation 11):

(11) $$\begin{array}{l} LL_{\mathrm{PE}}=\left(1-\frac{UL_{\mathrm{AR}}}{0.0788}\right)100\%\\ UL_{\mathrm{PE}}=\left(1-\frac{LL_{\mathrm{AR}}}{0.0788}\right)100\%\left[\left(1-\frac{0}{0.0788}\right)100\%=100\%\right]\; \end{array}$$

The above method for determining a confidence interval for PE assumes a constant estimated AR (7.88%). This assumption does not take into account additional variability had the malaria exposure during Study 033 been estimated based on results from a placebo control arm (with a given sample size). A sensitivity analysis was conducted (Table 4) to determine the effect of assuming a constant estimated attack rate on the resulting 95% lower confidence limit for the protective efficacy of tafenoquine [LL(PE_Tq_)]. Hypothetical estimated attack rates (AR_Pl_) from 1% -10% and sample sizes (n_PL_) from 10–1000 were considered. Combinations of AR_Pl_ and n_Pl_ were selected to give integer values for the corresponding number of placebo cases (n_PL_ × AR_PL_). All calculations assumed no observed cases in the tafenoquine arm and a sample size (ITT) of n = 490 (Study 033 results). Over the range of AR_Pl_ considered (1%-10%) the estimate of the lower limit of the 95% confidence interval (one-sided) for PE_Tq_ did not vary substantially with changes in sample size (<1% change, see Table 4). The minimal effect of sample size on LL(PE_Tq_) is because there were no observed malaria cases and the sample size in the tafenoquine arm employed in Study 033 was large (ITT n = 490). For a given AR_Pl_ and n_Pl_, Koopman's (score test) method was used for computing the lower 95% limit for PE. StatXact (vs. 9.0) was used and the confidence level was set to 90% (gives lower limit of a one-sided 95% interval and is the convention used when the point estimate for PE = 100%).

## Results

The observed attack rates for *P. falciparum* and *P. vivax* are tabulated for each ADF deployment in Table 1. All malaria species attack rates for 2RAR, 3RAR and 5/7 RAR were 10.13%, 11.20% and 6.71%, respectively (Table 1). All species attack rates were substantially lower for subsequent deployments (including 1 RAR), ranging from 0.53-1.62% (Table 1). The aggregate *P. falciparum* and *P. vivax* attack rates during the 1999/2000 deployments were 7.88% and 1.15%, yielding a *P. falciparum* to *P. vivax* species ratio of 0.146 (Table 2). For the 1 RAR deployment from 25 October 2000 to 25 April 2001, eight post-deployment *P. vivax* cases were observed amongst a deployed population of 723, yielding an observed post-deployment relapse rate of 1.11%.

All eight *P. vivax* cases following the 1RAR deployment were amongst individuals recruited for Study 033 (note that not all 723 1RAR soldiers were recruited for Study 033). Thus, the observed post-deployment *P. vivax* relapse rate during Study 033 was 1.23% (8/651). The number of *P. vivax* relapses observed (8) was higher than reported previously [6]. This was a consequence of extending the observation period from six months (per the study design) to 12 months due to additional data reported to the ADF’s CMR. The all malaria attack rate during the prophylactic phase of Study 033 was estimated to be 7.88% (see summary in Table 3). The CMR contained no records of malaria cases amongst 20 of 21 withdrawn subjects who remained in the ADF for twelve months following Study 033. A single withdrawn subject, randomized to the mefloquine arm, remained in the ADF for 11 months following Study 033. The CMR also contained no record of that subject having contracted malaria during this period.

In Study 033, of 490 ITT subjects given tafenoquine and 161 ITT subjects given mefloquine, no symptomatic cases of malaria were observed during the prophylactic phase ([6], see Table 5). None of the subjects listed as withdrawn from the study in the FCSR were found to have contracted malaria during the prophylactic phase. Therefore, the protective efficacy of both regimens was 100%. Utilizing the all species malaria attack rate of 7.88% during the prophylactic phase of the study as the estimated attack rate, the lower limits of the 95% confidence interval (one-sided, as outlined in Methods) for the protective efficacy of tafenoquine and mefloquine were 93% and 79%, respectively (Table 5).

**Table 5:** Protective efficacy (and confidence interval) for mefloquine and tafenoquine for malaria prophylaxis in non-immune Australian soldiers deployed to Timor Leste for six months in 2000/2001 (an attack rate of 7.88% was assumed)

	**Results during the prophylactic phase of Study 033**	**Protective efficacy (PE) during prophylactic phase of Study 033**				
Drug	ITT Population	Malaria cases	Subjects lost to follow-up	Drug attack rate (%) (95% CI)*	PE (%)**	95% CI***
Mefloquine	161	0	0	0 (0, 0.549)	100	79 - 100
Tafenoquine	490	0	0	0 (0, 1.653)	100	93 – 100

*Confidence interval (CI) for the attack rate (AR) is based on Wilson (score) method for proportions. In the special case when AR = 0 (0/n), the upper limit corresponds to a one-sided upper 95% limit. For AR = 0, the lower limit is set to zero.

**PE = [1 – drug AR/0.0788)]100. For zero cases, PE = 100%.

***95% CI for protective efficacy (PE) obtained from corresponding limits for the drug attack rates. The lower limit for PE = [1- AR upper limit/0.0788] x 100. The upper AR limits for tafenoquine (0/490) and mefloquine (0/161) are 0.549% and 1.653%. For PE = 100%, the upper limit is set to 100%.

## Discussion

This paper describes methodology for calculating protective efficacy when there is no concurrent placebo control arm data. Using this methodology, it was possible to demonstrate that tafenoquine exhibited a protective efficacy of 100% with a 95% lower confidence limit of 93% while mefloquine exhibited a protective efficacy of 100% with a 95% lower confidence limit of 79%. The lower value of the lower limit for mefloquine is due to the smaller sample size (161 *vs* 490). These calculations are based on a retrospective estimate of an attack rate of 7.88%. That attack rate was calculated based on the following assumptions: (i) that the *P. falciparum:P. vivax* species ratio in the Study 033 was the same as that directly observed in Australian soldiers in the 1999/2000 deployment, (ii) that the anti-relapse effectiveness of primaquine at the dose used in Study 033 was 69.5%, and (iii) that the anti-relapse efficacy of the tafenoquine dose used in Study 033 was 86.3%. These assumptions are conservative for the reasons outlined in the following paragraphs. Therefore, the attack rate reported in this study is likely to be an under-estimate.

The utilization of a *P. falciparum:P. vivax* ratio based on observed cases in Australian soldiers in 1999/2000 is the most conservative of the reasonable alternatives. It could have been assumed that there was no *P. falciparum* malaria during the 1 RAR deployment, since none was directly observed. Alternatively, the higher *P. falciparum:P. vivax* ratios observed in the unprophylaxed civilian population living close to garrisoned Australia soldiers during Study 033 (~1:1) or the ~2:1 ratio observed for malaria cases originating amongst Australian soldiers in Timor Leste during the 1999–2000 deployment [7,12] could have been used. Assuming no exposure to *P. falciparum* would not have been reasonable given that there was clearly transmission of *P. falciparum* amongst the civilian population living close to the 1 RAR garrison positions [7]. Assuming *P. falciparum:P. vivax* ratios of 1:1 or 2:1 would imply attack rates of 14% and 22% respectively. However, this would have assumed either parity in malaria exposure between unprophylaxed civilians and soldiers, or that compliance with doxycycline and primaquine prophylaxis could be estimated in the absence of data (remember that in Study 033 compliance with medications was recorded whereas in the 1999–2000 deployment it was not). While these assumptions may be defensible, the more conservative of the reasonable approaches was taken.

Using data from John *et al.*[15], it was assumed that the anti-relapse efficacy of the primaquine regimen used in Study 033 was 69.5%. This is likely to have been an under-estimate since the dose used in Study 033 (5.2 mg/kg) is higher than that (2.5 - <5 mg/kg) for which John *et al.* provide data. It was decided to use effectiveness data for low dose primaquine (2.5 - < 5 mg/kg) because there are only two studies in the literature which have compared relapse rates for a blood schizonticidal drug with and without high dose primaquine (> 5 mg/kg). Neither of these is directly analogous to Study 033. Baird *et al.*[22] reported that the anti-relapse efficacy of 10 mg/kg primaquine in combination with a standard chloroquine regimen in Irian Jaya was 82.3% relative to chloroquine alone after 28 days follow-up. However, the higher dose (10 mg/kg v 5.2 mg/kg in Study 033), short follow-up period (one month versus 12 months in Study 033) and the presence of chloroquine-resistance *P. vivax* in Irian Jaya (recurrences may be recrudescences not true relapses) are confounding. Leslie *et al.*[23] reported a higher anti-relapse efficacy for high dose primaquine in Pakistan. However, reliance on a single study from South Asia would have been inappropriate since recurrence rates following primaquine administration may be lower there than in South East Asia.

The assumed anti-relapse efficacy of tafenoquine of 86.3% was calculated using data for a prophylactic regimen of tafenoquine that was different from that used in Study 033 [4]. This is likely to have been an under-estimate since pharmacokinetic modeling studies suggest that this regimen (400 mg per day for three days followed by monthly maintenance doses) generates lower overall exposure levels than the Study 033 regimen despite the higher loading dose (Dow *et al*., unpublished observations). The calculations also assume similar *P. vivax* relapse rates and susceptibility to tafenoquine in Thailand and Timor Leste. This is reasonable given the long follow-up times in both the Walsh study and Study 033, and the lack of evidence suggesting any regional differences in susceptibility to 8-aminoquinolines in the western Pacific.

Mefloquine is an effective anti-malarial drug and remains the standard of care for weekly malaria prophylaxis where this is justified by the risk:benefit context. The reported efficacy of mefloquine prophylaxis in malaria naïve individuals is broadly similar to the efficacy of tafenoquine reported in this and prior clinical reports. In U.S. Peace Corps volunteers stationed in East Africa, where the majority of *P. falciparum* strains were chloroquine-resistant, the protective efficacy (95% CI) of mefloquine prophylaxis relative to chloroquine and chloroquine/proguanil prophylaxis was 94% (86-97%) and 86% (67-94%) respectively [24]. Presumably these also represent under-estimates of the true efficacy of mefloquine because both chloroquine and chloroquine-proguanil presumably have greater efficacy than placebo. In European tourists travelling to East Africa, the protective efficacy of mefloquine prophylaxis relative to no malaria prophylaxis was 94% [25].

The protective efficacy of tafenoquine and mefloquine during the deployment period in Study 033 was 100% since no symptomatic cases of malaria were observed. This was lower than the level of prophylactic efficacy of the same drugs (~86% for both drugs) observed amongst semi-immune residents of Ghana in one of the Phase II studies [2]. This seems counter-intuitive given the conventional wisdom that prophylactic anti-malarials should be more effective in non-immunes than semi-immunes due to the presumably enhancing effect of immunity in the latter group. However, it is not known whether this hypothesis is correct and there has not been a systematic review of the literature addressing this question. Also, there is a paucity of placebo-controlled field studies that have definitively determined the prophylactic efficacy of approved regimens in non-immune travellers since these are very challenging to perform. In the specific case of the Ghana [2] and Study 033, it is reasonable to speculate that the apparent differences in efficacy may be due to false positive microscopy. Even in a high attack rate setting, small rates of false positive microscopy (<1%) may result in an underestimate of true prophylactic efficacy that is of the order of magnitude of the difference in efficacy observed between the Ghana and Study 033 [26]. Furthermore, in a study involving non-immunes, a true malaria case, whether correctly or incorrectly diagnosed using microscopy, is likely to be recognized as such by the study team because it will be symptomatic. This is less likely to be the case in semi-immunes where it is routine to detect parasites by microscopy in individuals who are asymptomatic.

The conclusion that prophylactic efficacy of tafenoquine and mefloquine was 100% in Study 033 applies only to the period of time between the first dose of the drug and the first dose of post-deployment medication. In the case of mefloquine the persisting risk of a post-deployment case of *P. falciparum* malaria from a late deployment exposure is managed through administration of additional weekly doses of the drug for four weeks, while the risk of *P. vivax* relapses is managed through administration of primaquine (as in Study 033). In the case of tafenoquine, it is known that symptomatic failures occur when plasma levels of the drug fall below 80 ng/ml [27]. Therefore, it is anticipated that additional administration of tafenoquine will be required post-deployment to manage the residual risk of *P. falciparum* cases, although the precise regimen has not been determined. Based on open-label clinical studies [16], a residual risk of post-deployment *P. vivax* relapses of approximately the same magnitude as primaquine can be anticipated.

The population that may benefit the most from drugs for malaria prophylaxis are non-immune individuals deployed or travelling to areas with endemic malaria, although in some cases prophylaxis may also have considerable utility for malaria control efforts in endemic countries. In the context of a clinical study to determine the protective efficacy of a new prophylactic anti-malarial in non-immune individuals, it may be very challenging to directly determine an attack rate if the use of a placebo is not feasible. During a military engagement, placebo control arms are generally not used because of their possible impact on mission effectiveness. As argued elsewhere [5], the use of placebo in non-immune individuals more generally is acceptable if it does not expose study participants to a substantial risk of severe illness or death. This is feasible in Phase I challenge studies but may not be in a deployed setting for military or civilian participants. In the specific context of a prophylaxis study involving a deploying military force, if preparations for execution of the study are conducted rapidly there may be insufficient time to adequately document malaria exposure for two prior years as required by the FDA guidance [11]. One approach to determining a well-characterized attack rate would be to radically cure a cohort of civilians resident in the same area, and prospectively document new infections. Necessarily this assumes that civilian and military exposure to malaria is the same. The method described here, with different inherent assumptions, whereby an attack rate during the prophylactic phase is estimated based on adjustment of the observed post-deployment *P. vivax* relapse rate during follow-up to account for the anti-relapse efficacy of the study drugs, is an alternative approach that could be considered.

## Conclusions

This study retrospectively determined a conservative estimate of 7.88% for the all species malaria attack rate to which Australian Defence Forces personnel on peacekeeping duties in Timor Leste were exposed during their participation in a Phase III prophylaxis study to evaluate the prophylactic efficacy of tafenoquine and mefloquine. The prophylactic efficacy of mefloquine and tafenoquine during the period between administration of the first prophylactic dose and the first dose of post deployment medication was 100% (93-100%) and 100% (79-100%) respectively. The methodology employed to determine an attack retrospectively in the absence of placebo control arm should be considered when planning future prophylactic studies involving malaria naïve subjects in which it is not feasible to include a placebo control arm.

## Competing interests

The authors have a personal and professional interest in ensuring the regulatory approval of tafenoquine for malaria prophylaxis but no competing interests.

## Authors’ contributions

GSD led the technical team that performed the analysis and wrote the manuscript. MR extracted records from the Central Malaria Registry and conducted the analysis of attack rates from prior deployments. WFM and DT performed the statistical analyses. BS and DS provided valuable perspective on the clinical aspects of malaria, methodological aspects of clinical trial and product history with tafenoquine. All authors read and approved the final manuscript.
